# A full-cycle integrated management model for esophageal cancer in county health systems: effects on care processes, clinical outcomes, quality of life, and cost-utility

**DOI:** 10.3389/fonc.2026.1821733

**Published:** 2026-07-27

**Authors:** Jianjia Xiao, Limei Ye, Jinduan Zhou, Mingmei Li, Yuchao Sang, Dongqin Huang, Qiaoqin Lin, Weihong Chen

**Affiliations:** 1 Department of Tumor Prevention and Control Center, Anxi County Hospital, Quanzhou, China; 2 Department of Medical Oncology, Anxi County Hospital, Quanzhou, China; 3 Department of Clinical Medical Research Center, Anxi County Hospital, Quanzhou, China; 4 Anxi County Guanqiao Hospital, Quanzhou, China

**Keywords:** controlled pilot study, county health services, esophageal cancer, full-cycle management, integrated care, three-tier service network

## Abstract

**Background:**

Esophageal cancer (EC) remains a major public health challenge in high-incidence, resource-limited county settings, where fragmented services often lead to delayed diagnosis, suboptimal multidisciplinary care, and poor continuity of follow-up. We developed a county-adapted, integrated “prevention–screening–treatment” model with full-cycle closed-loop management and evaluated its effectiveness.

**Methods:**

We conducted a controlled pilot study in Anxi County, Fujian Province, China. The intervention was delivered through a county–township–village three-tier service network supported by a digital management platform and standardized quality-of-life (QoL) assessment. Two hundred adults with pathologically confirmed EC were enrolled and assigned (non-randomly, based on enrollment in the program pathway at first presentation) to the intervention group receiving the integrated full-cycle management model (n=100) or to a control group receiving usual county-level care (n=100). Primary outcomes included diagnostic delay, multidisciplinary team (MDT) participation, standardized follow-up, and survival outcomes. Secondary outcomes included treatment adherence, complications, QoL (EORTC QLQ-C30 and QLQ-OES18), and cost-utility (cost per quality-adjusted life year, QALY). Between-group comparisons used χ² tests and t tests; longitudinal QoL used repeated-measures analysis; and survival used Cox regression.

**Results:**

Compared with controls, the intervention group had shorter diagnostic delay (46.8 ± 13.2 vs 88.5 ± 17.6 days; p < 0.001), higher MDT participation (91.0% vs 34.0%; p < 0.001), higher standardized follow-up (86.0% vs 32.0%; p < 0.001), and lower loss to follow-up (4.0% vs 17.0%; p = 0.001). One-, two-, and three-year overall survival was higher in the intervention group (88.0%, 75.0%, and 67.0%) than in controls (71.0%, 57.0%, and 55.0%). QoL improved more over time in the intervention group, with significant group-by-time interactions for key functional and symptom domains (all p < 0.001). Direct medical and indirect costs were lower, while QALYs were higher (0.696 ± 0.127 vs 0.602 ± 0.119; p < 0.001), resulting in a lower cost per QALY.

**Conclusions:**

A county-adapted, integrated full-cycle EC management model supported by a three-tier service network, digital tools, and standardized QoL measurement improved care processes, outcomes, and cost-utility. This approach appears feasible and potentially scalable to similar high-incidence county settings.

## 1 Introduction

Esophageal cancer (EC) remains a major global public health threat because of its aggressive biology, late clinical presentation, and substantial mortality burden. The latest comprehensive global estimates from GLOBOCAN 2022 indicate that cancer overall continues to impose a heavy and uneven burden across world regions, with marked disparities by development level and health-system capacity (1). Within this landscape, EC is consistently recognized as one of the most lethal malignancies, contributing disproportionately to cancer deaths relative to its incidence in many countries (2).

Importantly, EC burden and etiology vary substantially by geography and histologic subtype. Esophageal squamous cell carcinoma (ESCC) predominates across the so-called “Asian esophageal cancer belt” and many parts of China, whereas esophageal adenocarcinoma (EAC) has become the dominant subtype in several Western countries (3–5). These subtype differences reflect divergent risk-factor profiles and prevention opportunities: ESCC is more strongly linked to tobacco and alcohol exposure, dietary and nutritional factors, and thermal injury, whereas EAC is more closely associated with reflux disease and obesity-related pathways (3, 6).

China bears a particularly large share of the global EC burden. Recent GLOBOCAN 2022–based analyses estimate ~224, 012 new EC cases and ~187, 467 deaths in China in 2022, underscoring the scale of the challenge and the need for regionally tailored control strategies (7). In high-incidence coastal and rural areas, long-standing lifestyle patterns and dietary exposures (e.g., frequent intake of preserved foods and thermal injury from hot beverages) have been repeatedly implicated as contributors to ESCC risk, mirroring risk-factor constellations observed in other global high-incidence settings (8–11).

From a clinical standpoint, EC prognosis is strongly stage-dependent. When detected at an early stage, endoscopic submucosal dissection (ESD) can achieve a 5-year relative overall survival rate of 99.0%, with a cause-specific survival rate of 100% at a median follow-up of 72.9 months (12, 13). However, population-based 5-year survival for EC remains poor, ranging from approximately 15–20% in most countries (13). Moreover, data from the SEER database show that the most common stage at presentation for both adenocarcinoma (36.9%) and squamous cell carcinoma (26.8%) is stage IV (2, 14–16). Highlighting the challenge of early detection in routine practice.

These challenges are amplified in resource-limited county (rural) settings and, more broadly, in many low- and middle-income country (LMIC) contexts. Structural constraints such as limited endoscopy capacity and trained personnel, delayed referral pathways, restricted access to multidisciplinary decision-making, and fragmented post-treatment follow-up can produce a “late diagnosis, repeated referrals, and weak continuity” pattern across the cancer care continuum. Evidence from China and other regions emphasizes that effective EC control requires not only technical advances in treatment but also system-level measures that improve screening organization, referral efficiency, and longitudinal management within real-world health systems (6, 17).

Screening and early diagnosis are especially pivotal. Endoscopy is widely regarded as the key modality for ESCC detection in high-risk populations, yet translating endoscopic screening into routine county practice is difficult due to cost, workforce, and infrastructure constraints. Recent randomized or trial-based evidence from high-risk areas in China suggests that invitation to endoscopic screening can reduce upper gastrointestinal cancer mortality, supporting the public health value of organized screening when paired with effective downstream management (18, 19). These data also imply that screening impact may depend on how well the system ensures timely diagnostic confirmation, standardized treatment linkage, and follow-up, i.e., the “screen–diagnose–treat–monitor” chain must function as a coordinated whole rather than as isolated steps.

In parallel, modern cancer control increasingly emphasizes patient-centered outcomes and survivorship quality, not only survival. The EORTC QLQ-C30 and the esophageal-specific QLQ-OES18 are internationally validated instruments for monitoring health-related quality of life (QoL) and symptom burden in oncology and EC populations (20, 21). Nevertheless, in many county-level workflows these tools are not routinely integrated into clinical pathways, limiting the ability to quantify symptom trajectories, evaluate supportive care effectiveness, and compare performance across settings.

Taken together, the above gaps highlight a critical implementation bottleneck: policies and technical recommendations may exist, but many county systems still lack an operational “bridge” that integrates prevention, screening, diagnosis, treatment, rehabilitation, and follow-up into a coherent, measurable, and sustainable service framework supported by digital tools and standardized evaluation. Against this backdrop, developing a county-adapted integrated model with full-cycle closed-loop management may offer a pragmatic approach to improving EC outcomes in high-incidence, resource-constrained settings. Therefore, we developed a model delivered through a county–township–village network, supported by a digital management platform, and anchored in standardized QoL assessment; we evaluated its effectiveness in improving care processes, clinical outcomes, QoL, and cost-utility among patients with EC.

## 2 Methods

### 2.1 Study design and setting

This controlled pilot study was conducted in Anxi County, Fujian Province, China, a county-level region with a high burden of EC. The intervention consisted of an integrated “prevention–screening–treatment” model with full-cycle closed-loop management. The study followed the principles of the Declaration of Helsinki and was approved by the Medical Ethics Committee of Anxi County Hospital (approval No. 20240017). All participants provided written informed consent.

### 2.2 Participants

Eligible participants were adult patients with pathologically confirmed EC who met the diagnostic criteria of the national “Esophageal Cancer Screening and Early Diagnosis and Treatment Program (2024 edition)”. Key inclusion criteria were an expected survival of at least 6 months, residency within the county medical consortium catchment area, and willingness to participate and complete follow-up and questionnaire assessments. Exclusion criteria included severe psychiatric or cognitive disorders precluding participation, severe systemic diseases expected to prevent completion of follow-up, withdrawal from the intervention, or incomplete clinical data.

### 2.3 Sample size and grouping

A total of 200 EC patients were enrolled, with 100 assigned to the intervention group and 100 to the control group. As this is a pilot study, no formal sample size calculation was performed; the sample size was based on feasibility and the operational capacity of the county medical consortium. Baseline characteristics (e.g., sex, age, TNM stage, Karnofsky Performance Status [KPS], comorbidities, and treatment modality) were comparable between groups.

### 2.4 Intervention: integrated full-cycle closed-loop management

#### 2.4.1 Group allocation and follow-up

The intervention was implemented as a real-world service-delivery program within the county medical consortium; therefore, group assignment was non-random. Patients were allocated according to whether they entered the integrated full-cycle management pathway at their first presentation. Those managed within the integrated pathway comprised the intervention group, whereas patients receiving routine county-level management outside the integrated framework comprised the control group. All participants were followed for up to 3 years to ascertain survival and recurrence/metastasis outcomes. Health-related quality of life (QoL) was assessed at baseline and at 6, 12, and 24 months using standardized questionnaires.

#### 2.4.2 Intervention components

**Figure 1:**
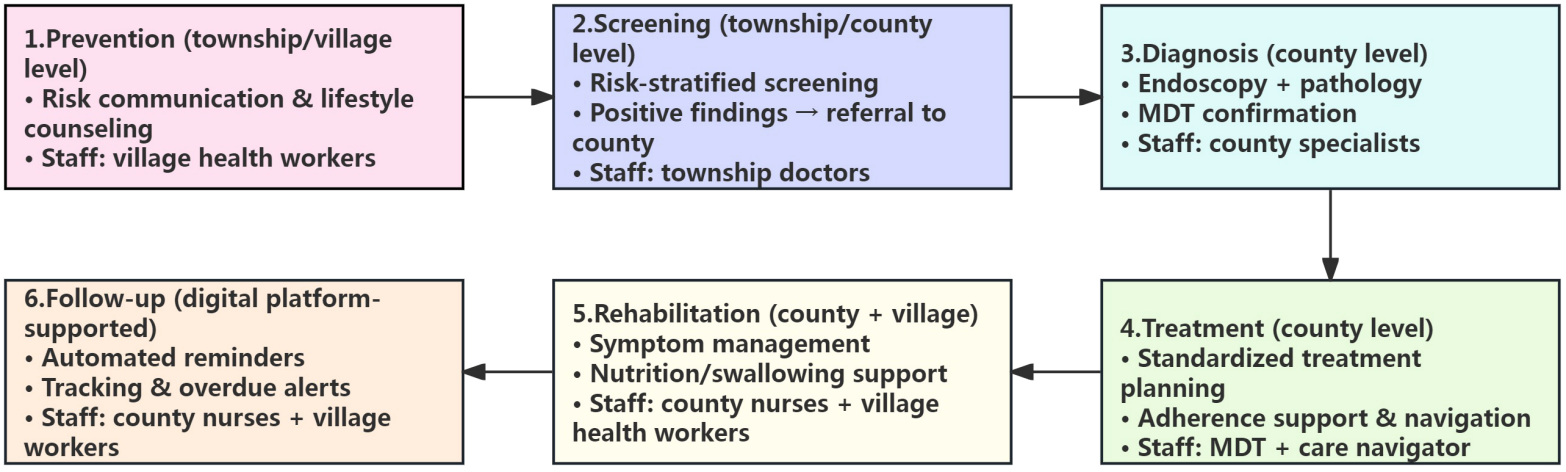
Flow diagram of the integrated full-cycle closed-loop management model. MDT, multidisciplinary team. The model operates through a county–township–village three-tier network supported by a digital management platform. Staff responsibilities and entry/exit points are indicated for each stage.

The model used a county–township–village three-tier network and a digital management platform (**Figure 1**). Patients entered at township/village level via risk assessment; positive findings triggered electronic referral to county hospital for endoscopic screening and MDT diagnosis. The MDT formulated treatment plans with adherence support. Post-treatment, village health workers conducted monthly symptom checks, while county nurses managed scheduled re-examinations. The digital platform automated reminders and tracked overdue tasks. Routine QoL monitoring (EORTC QLQ-C30/QES18) was embedded. The control group received usual county care without these integrated components. In the three-tier network, village health workers perform risk assessment and monthly follow-up, township doctors conduct screening and referral, and county specialists provide diagnosis, MDT treatment, and oversight.

#### 2.4.3 Control condition

Patients in the control group received usual county-level care according to routine clinical practice, without the integrated three-tier network, default time-bound MDT workflow, digital closed-loop follow-up, or routine integration of QoL measures into care management.

### 2.5 Outcomes

Primary implementation and effectiveness outcomes included diagnostic delay (days from symptom onset to confirmed diagnosis), MDT participation, standardized follow-up, and adherence to scheduled re-examinations. Clinical outcomes included 1–3-year overall survival, 3-year disease-free survival, recurrence/metastasis rates, and complication rates. Patient-reported outcomes included longitudinal QoL assessed at baseline, 6 months, 12 months, and 24 months using the EORTC QLQ-C30 and symptom domains of the QLQ-OES18 (20, 22). Health economic outcomes included direct medical costs (e.g., inpatient and treatment costs), indirect costs (e.g., transportation and productivity loss), QALYs, and cost per QALY.

### 2.6 Cost-utility analysis

This analysis was conducted from a healthcare system perspective, including direct medical costs and indirect costs (transportation and productivity loss). Costs were collected in Chinese Yuan (CNY) from medical records and participant-reported expenditures. QALYs were estimated by combining follow-up time with health utility weights derived from QoL data using a published mapping approach from EORTC QLQ-C30 scores to preference-based utility values, and by calculating the area under the utility curve over follow-up.

### 2.7 Statistical analysis

**Figure 2:**
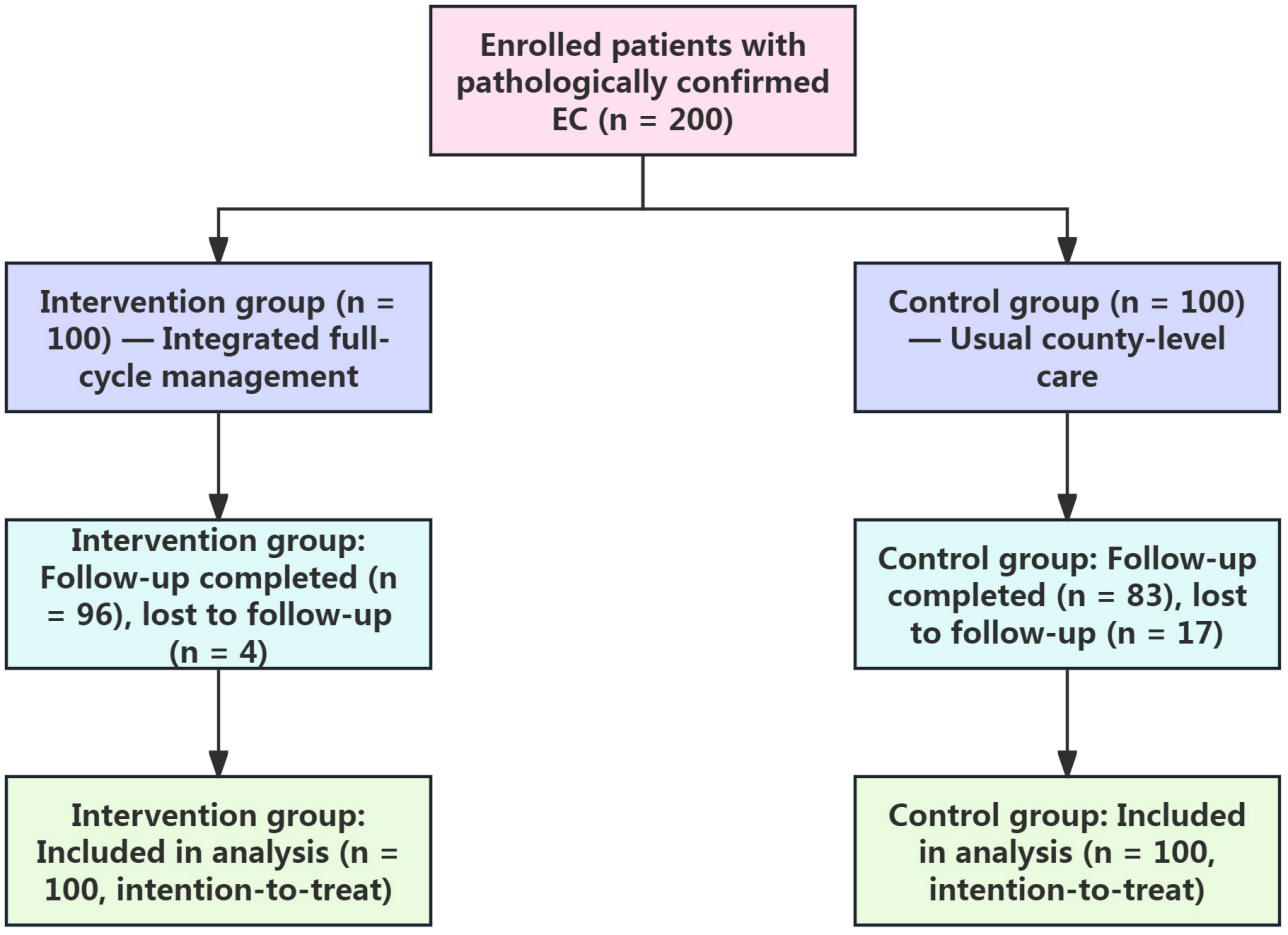
Participant flow diagram. A total of 200 patients were enrolled, with 100 assigned to the intervention group and 100 to the control group. Follow-up and analysis numbers are shown.

Analyses were performed using SPSS 26.0. Categorical and continuous variables were compared using χ² tests and t tests, respectively. For longitudinal QoL outcomes, a linear mixed-effects model with unstructured covariance assessed group-by-time interactions. Missing QoL data were minimal (<5%) and assumed missing at random; no imputation was performed. Cox regression was used for survival analysis; the proportional hazards assumption was verified (Schoenfeld residuals, *p* > 0.05). Multivariable models adjusted for TNM stage, KPS, comorbidity burden, treatment modality, sex, and age, selected *a priori* based on clinical relevance and univariate associations (*p* < 0.10). Missing baseline data were minimal (<5%); complete-case analysis was performed. Two-sided *p* < 0.05 was considered significant. A participant flow diagram is presented in **Figure 2**.

## 3 Results

### 3.1 Participant characteristics and baseline comparability

**Figure 2** shows the participant flow through the study. Briefly, 200 patients with pathologically confirmed EC were included (n=100 per group). The two groups were well balanced at baseline, with no significant differences in key demographic variables, disease stage, performance status, comorbidity profile, or treatment modality (all p > 0.05; **Table 1**). Males comprised 57.0% of the intervention group and 54.0% of the control group (p=0.501). Mean age was 64.5 ± 8.2 vs 65.3 ± 9.1 years (p=0.531). TNM stage distribution was similar (p=0.645): stage III was most common (48.0% vs 51.0%), followed by stage IV (29.0% vs 28.0%) and stage I–II (23.0% vs 21.0%). KPS was comparable: 65.0% vs 63.0% had KPS 90–100 (p=0.321). Comorbidity prevalence was similar between groups: hypertension (38.0% vs 40.0%, p=0.687), diabetes (24.0% vs 26.0%, p=0.724), and coronary heart disease (15.0% vs 14.0%, p=0.821). Treatment modality distribution was also well matched (p=0.789): chemo-/radiotherapy (45.0% vs 48.0%), surgery (36.0% vs 33.0%), immuno-/targeted therapy (12.0% vs 11.0%), and conservative/no treatment (7.0% vs 8.0%). The integrated full-cycle management pathway is detailed in **Figure 1**.

**Table 1:** Baseline characteristics of patients with esophageal cancer (n = 100 per group).

Characteristic	Intervention (n=100)	Control (n=100)	χ²/F	*p* value
Sex [n (%)]			0.452	0.501
Male	57 (57.0)	54 (54.0)		
Female	43 (43.0)	46 (46.0)		
Age (years), mean ± SD	64.5 ± 8.2	65.3 ± 9.1	0.628	0.531
TNM stage [n (%)]			0.876	0.645
Stage I–II	23 (23.0)	21 (21.0)		
Stage III	48 (48.0)	51 (51.0)		
Stage IV	29 (29.0)	28 (28.0)		
KPS category [n (%)]			0.987	0.321
0–80	35 (35.0)	37 (37.0)		
90–100	65 (65.0)	63 (63.0)		
Comorbidities [n (%)]				
Hypertension	38 (38.0)	40 (40.0)	0.162	0.687
Diabetes	24 (24.0)	26 (26.0)	0.125	0.724
Coronary heart disease	15 (15.0)	14 (14.0)	0.051	0.821
Treatment modality [n(%)]			1.053	0.789
Surgery	36 (36.0)	33 (33.0)		
Chemo-/radiotherapy	45 (45.0)	48 (48.0)		
Immuno-/targeted therapy	12 (12.0)	11 (11.0)		
Conservative/no treatment	7 (7.0)	8 (8.0)		

### 3.2 Care pathway performance and patient engagement

As shown in **Table 2**, the intervention group had shorter diagnostic delay (46.8 ± 13.2 vs 88.5 ± 17.6 days, p<0.001), higher MDT participation (91.0% vs 34.0%, p<0.001), higher standardized follow-up (86.0% vs 32.0%, p<0.001), higher adherence to scheduled re-examinations (90.0% vs 55.0%, p<0.001), and lower loss to follow-up (4.0% vs 17.0%, p=0.001). **Table 3** shows better patient engagement in the intervention group: treatment adherence (95.0% vs 73.0%, p<0.001), rehabilitation implementation (87.0% vs 41.0%, p<0.001), health education awareness (93.0% vs 62.0%, p<0.001), and unhealthy lifestyle improvement (85.0% vs 48.0%, p<0.001).

**Table 2:** Comparison of diagnostic timeliness and follow-up performance indicators between the intervention and control groups.

Indicator	Intervention (n=100)	Control (n=100)	Statistic	*p* value
Diagnostic delay (days), mean ± SD	46.8 ± 13.2	88.5 ± 17.6	t=18.742	<0.001
MDT participation [n(%)]	91 (91.0)	34(34.0)	χ²=76.364	<0.001
Standardized follow-up [n(%)]	86 (86.0)	32(32.0)	χ²=64.571	<0.001
Adherence to scheduled re-examinations [n(%)]	90 (90.0)	55(55.0)	χ²=32.444	<0.001
Loss to follow-up [n(%)]	4 (4.0)	17(17.0)	χ²=10.667	0.001

**Table 3:** Comparison of treatment adherence, rehabilitation uptake, and health behavior indicators between the intervention and control groups [n (%)].

Indicator	Intervention (n=100)	Control (n=100)	χ²	p value
Adherence to treatment plan	95 (95.0)	73 (73.0)	18.778	<0.001
Rehabilitation implementation rate	87 (87.0)	41 (41.0)	42.240	<0.001
Health education awareness	93 (93.0)	62 (62.0)	26.786	<0.001
Unhealthy lifestyle improvement rate	85 (85.0)	48 (48.0)	31.360	<0.001

### 3.3 Survival, disease control, complications, and adjusted mortality risk

As shown in **Table 4**, the intervention group had higher overall survival at 1 year (88.0% vs 71.0%, p=0.001), 2 years (75.0% vs 57.0%, p=0.002), and 3 years (67.0% vs 55.0%, p=0.041), as well as higher 3-year disease-free survival (61.0% vs 44.0%, p=0.002). The recurrence/metastasis rate was lower in the intervention group (22.0% vs 38.0%, p=0.008), particularly local recurrence (12.0% vs 20.0%, p=0.048). **Table 5** shows fewer complications in the intervention group: overall incidence 17.0% vs 34.0% (p=0.002), with significant reductions in pulmonary infection (7.0% vs 15.0%, p=0.034), anastomotic leakage (3.0% vs 9.0%, p=0.040), and gastrointestinal bleeding (2.0% vs 7.0%, p=0.048). Multivariable Cox regression (**Table 6**) confirmed that the integrated model was independently associated with lower mortality risk (HR = 0.539, 95% CI 0.435–0.668, p<0.001). Advanced TNM stage (HR = 2.375), higher comorbidity burden (HR = 1.536), lower KPS (HR = 0.584), and no active treatment (HR = 0.684) were also independent predictors (all p<0.01).

**Table 4:** Overall survival and disease control outcomes (recurrence and metastasis) in the intervention and control groups [n (%)].

Indicator	Intervention (n=100)	Control (n=100)	Statistic	*p* value
1-year survival	88 (88.0)	71 (71.0)	χ²=11.025	0.001
2-year survival	75 (75.0)	57 (57.0)	χ²=9.211	0.002
3-year survival	67 (67.0)	55 (55.0)	χ²=4.167	0.041
3-year disease-free survival	61 (61.0)	44 (44.0)	χ²=9.683	0.002
Recurrence/metastasis rate	22 (22.0)	38 (38.0)	χ²=7.031	0.008
Local recurrence rate	12 (12.0)	20 (20.0)	χ²=3.914	0.048
Distant metastasis rate	10 (10.0)	18 (18.0)	χ²=3.175	0.075

**Table 5:** Treatment-related complications in the intervention and control groups [n (%)].

Complication	Intervention (n=100)	Control (n=100)	χ²	p value
Pulmonary infection	7 (7.0)	15 (15.0)	4.507	0.034
Anastomotic leakage	3 (3.0)	9 (9.0)	4.237	0.040
Gastrointestinal bleeding	2 (2.0)	7 (7.0)	3.914	0.048
Electrolyte disorder	4 (4.0)	8 (8.0)	2.105	0.147
Other complications	1 (1.0)	5 (5.0)	3.004	0.083
Overall incidence	17 (17.0)	34 (34.0)	9.333	0.002

**Table 6:** Multivariable Cox regression identifying independent predictors of 3-year mortality in esophageal cancer.

Variable	Coding	β	SE	Hazard ratio (HR)	95% CI	*p* value
Sex	Male=1, Female=0	0.118	0.102	1.125	0.923~1.372	0.234
Age (years)	Continuous	0.007	0.006	1.007	0.996~1.018	0.215
TNM stage	Stage I–II=0, Stage III = 1, Stage IV = 2	0.864	0.132	2.375	1.896~2.978	<0.001
KPS category	0-80 = 0, 90-100 = 1	-0.538	0.112	0.584	0.478~0.713	<0.001
Number of comorbidities	0-1 = 0, ≥2 = 1	0.429	0.121	1.536	1.243~1.897	<0.001
Management model	Usual care=0, full-cycle model=1	-0.618	0.126	0.539	0.435~0.668	<0.001
Treatment modality	Conservative=0, active treatment=1	-0.379	0.142	0.684	0.532~0.879	0.003

### 3.4 Patient-reported quality of life and symptom burden

Longitudinal QoL was assessed using EORTC QLQ-C30 (**Tables 7**, **8**) and QLQ-OES18 (**Table 9**). As shown in **Table 7**, the intervention group showed greater improvements in all functional domains and global health status over 24 months (all group-by-time p < 0.001). At 24 months, the intervention group had higher scores for physical (81.2 ± 7.8 vs 69.8 ± 9.3), role (78.5 ± 9.5 vs 62.9 ± 10.9), emotional (79.3 ± 8.9 vs 66.8 ± 10.2), and global health (74.8 ± 9.2 vs 58.9 ± 10.4).**Table 8** shows greater symptom reduction in the intervention group (all p < 0.001). At 24 months, the intervention group reported lower fatigue (23.9 ± 10.7 vs 36.8 ± 11.9), pain (22.1 ± 9.9 vs 33.7 ± 11.3), and nausea/vomiting (11.2 ± 9.1 vs 21.0 ± 10.5). **Table 9** shows better EC-specific outcomes in the intervention group: lower scores for dry mouth, coughing, pain when eating, dysphagia, and chest pain (all p < 0.001), and higher scores for ability to eat solid foods (61.8 ± 12.5 vs 41.5 ± 13.6) and enjoyment of eating (67.8 ± 11.3 vs 41.9 ± 12.9) (both p < 0.001).

**Table 7:** Longitudinal comparison of EORTC QLQ-C30 functional domain scores and global health status between the intervention and control groups.

Domain	Time point	Intervention (n=100)	Control (n=100)	F value	*p* value
Physical functioning	Baseline	65.1 ± 10.5	64.7 ± 10.8	28.452	<0.001
	6 months	71.8 ± 9.9	65.9 ± 10.3		
	12 months	77.5 ± 8.7	68.2 ± 9.8		
	24 months	81.2 ± 7.8	69.8 ± 9.3		
Role functioning	Baseline	58.3 ± 12.5	57.8 ± 12.7	25.683	<0.001
	6 months	66.9 ± 11.3	59.1 ± 11.9		
	12 months	74.2 ± 10.2	61.3 ± 11.4		
	24 months	78.5 ± 9.5	62.9 ± 10.9		
Emotional functioning	Baseline	62.1 ± 11.7	61.5 ± 11.9	22.847	<0.001
	6 months	69.8 ± 10.5	63.2 ± 11.1		
	12 months	75.6 ± 9.7	65.4 ± 10.6		
	24 months	79.3 ± 8.9	66.8 ± 10.2		
Global health status	Baseline	53.2 ± 12.0	52.7 ± 12.2	30.568	<0.001
	6 months	61.9 ± 10.7	55.1 ± 11.3		
	12 months	69.5 ± 9.9	57.3 ± 10.9		
	24 months	74.8 ± 9.2	58.9 ± 10.4		

**Table 8:** Longitudinal comparison of EORTC QLQ-C30 symptom scale scores between the intervention and control groups.

Domain	Time point	Intervention (n=100)	Control (n=100)	F value	*p* value
Fatigue	Baseline	42.3 ± 13.4	42.8 ± 13.6	24.753	<0.001
	6 months	34.8 ± 12.3	41.5 ± 12.9		
	12 months	28.2 ± 11.5	39.3 ± 12.4		
	24 months	23.9 ± 10.7	36.8 ± 11.9		
Pain	Baseline	39.5 ± 12.7	39.9 ± 12.9	21.364	<0.001
	6 months	32.1 ± 11.6	38.4 ± 12.2		
	12 months	26.3 ± 10.8	36.2 ± 11.8		
	24 months	22.1 ± 9.9	33.7 ± 11.3		
Nausea/vomiting	Baseline	26.1 ± 11.9	25.7 ± 12.1	18.542	<0.001
	6 months	18.3 ± 10.7	24.3 ± 11.4		
	12 months	13.8 ± 9.9	22.5 ± 11.0		
	24 months	11.2 ± 9.1	21.0 ± 10.5		

**Table 9:** EORTC QLQ-OES18 esophageal symptom scores at 24-month follow-up in the intervention and control groups.

Domain	Intervention (n=100)	Control (n=100)	t value	*p* value
Dry mouth	25.8 ± 9.9	42.7 ± 11.6	10.842	<0.001
Trouble with coughing	10.9 ± 8.6	28.3 ± 10.4	12.367	<0.001
Pain when eating	12.1 ± 9.0	32.1 ± 11.3	14.258	<0.001
Ability to eat solid foods	61.8 ± 12.5	41.5 ± 13.6	11.987	<0.001
Dysphagia	14.5 ± 9.6	38.2 ± 12.2	16.324	<0.001
Chest pain	12.3 ± 9.3	31.5 ± 11.4	13.571	<0.001
Enjoyment of eating	67.8 ± 11.3	41.9 ± 12.9	15.843	<0.001

### 3.5 Costs and cost-utility outcomes

As shown in **Table 10**, the intervention group had lower direct medical costs (67, 852.4 ± 15, 426.8 vs 80, 695.7 ± 18, 235.4 CNY, p<0.001), driven by reductions in inpatient costs, chemo-/radiotherapy costs, and medication costs (all p<0.001). Indirect costs were also lower in the intervention group (5, 789.6 ± 2, 118.5 vs 8, 864.3 ± 2, 845.7 CNY, p<0.001). The intervention group accrued higher QALYs (0.696 ± 0.127 vs 0.602 ± 0.119, p<0.001), resulting in a lower cost per QALY (97, 568.3 ± 12, 459.6 vs 134, 785.6 ± 15, 582.7 CNY/QALY, p<0.001).

**Table 10:** Comparison of health economic outcomes (costs, QALYs, and cost-utility) between the intervention and control groups.

Indicator	Intervention (n=100)	Control (n=100)	t value	*p* value
Direct medical cost (CNY)	67852.4 ± 15426.8	80695.7 ± 18235.4	5.487	<0.001
Total inpatient cost (CNY)	51968.7 ± 13125.9	63854.2 ± 15568.7	5.742	<0.001
Surgery cost (CNY)	28256.3 ± 8895.7	29874.5 ± 9432.6	1.248	0.213
Chemo-/radiotherapy cost (CNY)	18542.6 ± 6489.3	24156.8 ± 7789.5	4.876	<0.001
Medication cost (CNY)	12345.8 ± 4289.6	15478.3 ± 5098.4	4.562	<0.001
Indirect cost (CNY)	5789.6 ± 2118.5	8864.3 ± 2845.7	8.743	<0.001
Transportation cost (CNY)	1542.8 ± 886.5	2845.6 ± 1032.8	9.215	<0.001
Productivity loss (CNY)	3215.7 ± 1236.8	4956.8 ± 1547.9	8.964	<0.001
QALYs (years)	0.696 ± 0.127	0.602 ± 0.119	4.683	<0.001

### 3.6 Implementation acceptability: patient satisfaction and staff endorsement

As shown in **Table 11**, the intervention group reported higher patient satisfaction across all domains: screening services (89.2 ± 8.7 vs 72.1 ± 10.5), care pathway (90.8 ± 7.9 vs 75.3 ± 9.9), rehabilitation guidance (90.1 ± 8.3 vs 68.4 ± 11.3), and follow-up management (91.9 ± 7.6 vs 65.1 ± 12.2). Healthcare staff (n=50 per group) also showed greater acceptance of the intervention model for ease of operation (86.0% vs 51.0%), resource integration (89.0% vs 46.0%), and work efficiency improvement (84.0% vs 54.0%). The incremental cost-effectiveness ratio (ICER) was –136, 630 CNY per QALY, indicating that the intervention was dominant (lower cost, higher QALYs) compared with usual care.

**Table 11:** Comparison of patient satisfaction and healthcare staff acceptance between the intervention and control groups.

Indicator	Intervention	Control	Statistic	p value
Patient satisfaction (score, mean ± SD)				
Screening services	89.2 ± 8.7	72.1 ± 10.5	t=11.842	<0.001
Care pathway	90.8 ± 7.9	75.3 ± 9.9	t=10.753	<0.001
Rehabilitation guidance	90.1 ± 8.3	68.4 ± 11.3	t=14.268	<0.001
Follow-up management	91.9 ± 7.6	65.1 ± 12.2	t=16.847	<0.001
Healthcare staff acceptance [n(%)]	n=50	n=50		
Ease of operation	43 (86.0)	25 (51.0)	χ²=15.680	<0.001
Resource integration	44 (89.0)	23 (46.0)	χ²=21.632	<0.001
Work efficiency improvement	42 (84.0)	27 (54.0)	χ²=11.520	0.001

## 4 Discussion

In this county-based controlled pilot study conducted in a high-incidence esophageal cancer (EC) area, we designed, implemented, and evaluated a county-adapted “integrated prevention–treatment” model supported by full-cycle closed-loop management. Using a controlled cohort of 200 pathologically confirmed patients followed for three years, we found that the model was associated with consistent advantages over routine county-level management across multiple domains including shorter diagnostic delay, stronger continuity of care, improved clinical outcomes, better patient-reported quality of life, and lower healthcare costs. These findings are highly relevant given the substantial global burden of cancer and the persistent challenges of delivering timely, coordinated cancer care in resource-constrained settings (1).

A critical strength of this evaluation is the comparability of baseline characteristics between groups. Sex, age, TNM stage distribution, comorbidity profile, and treatment modalities did not differ significantly, which strengthens the internal validity of between-group comparisons. From an epidemiologic perspective, the sample profile aligns with the typical demographic and clinical presentation patterns seen in many EC cohorts, including older age and a substantial comorbidity burden, factors known to influence perioperative risk and long-term outcomes (23, 24). This representativeness supports cautious generalization to similar county-level high-incidence settings where EC prevention and control are challenged by late presentation, fragmented pathways, and limited specialty resources.

The most policy-relevant finding is the improvement in “prevention and control effectiveness, “ which in practice represents a system solution to fragmented service delivery. The intervention nearly halved the average time from symptom onset to confirmed diagnosis. This improvement is plausibly attributable to the combined impact of resource integration and process optimization. Upstream triage through a county–township–village three-tier network can reduce downstream congestion and shorten care-seeking-to-diagnosis intervals by shifting early recognition, first-contact assessment, and referral initiation closer to patients. At the same time, time-bound MDT workflows and digital referral/reminder functions can reduce “administrative latency” and strengthen guideline-concordant decision-making. MDT involvement has been associated with improved survival for esophageal cancer patients in large observational cohorts, supporting the plausibility that MDT-enabled coordination may translate process gains into better outcomes (25, 26).

Continuity of care, particularly follow-up, often represents the weakest link in rural cancer control, and the intervention yielded a marked improvement in this domain. The observed rise in standardized follow-up and reduced loss-to-follow-up likely reflect the synergy of technology enablement and responsibility decentralization, although this mechanism was not directly tested in the present study. Digital reminder and tracking strategies (including SMS-based and other automated reminders) have repeatedly been shown to improve appointment adherence and medical compliance across healthcare settings, providing supportive external evidence for the mechanism underlying follow-up improvements in our model (26). In parallel, the redistribution of certain longitudinal tasks (education, basic follow-up, data capture) to grassroots team is consistent with the broader evidence base on task-shifting/task-sharing strategies in low- and middle-income countries, where transferring selected responsibilities from specialists to trained non-physician or primary-care health workers can improve access and continuity for chronic disease management (27). Although cancer care differs from traditional NCD programs, our findings suggest that similar principles such as clear task delineation, supervision, and information support may also strengthen survivorship-oriented follow-up in county oncology pathways.

Improved processes translated into meaningful clinical benefits, reinforcing the effectiveness of the integrated model. The intervention group achieved higher 1–3-year survival rates, higher disease-free survival, and lower recurrence/metastasis. MDT-driven individualized planning may reduce both under-treatment and over-treatment and improve adherence to multidisciplinary, stage-appropriate pathways. This inference aligns with population-level evidence that MDT participation is associated with improved survival outcomes in EC (25, 26). Importantly, the multivariable Cox results in our study further support an independent association between the integrated model and reduced mortality risk after adjustment for key prognostic factors, which is consistent with the broader health-systems premise that redesigning care pathways can yield outcome benefits beyond patient and tumor biology alone.

The reduction in complications provides additional support for the model’s clinical value. Severe post-esophagectomy complications (e.g., pulmonary infection and anastomotic leakage) are among the most consequential drivers of prolonged hospitalization, readmission, and downstream mortality risk (28, 29). Modern perioperative optimization and standardized postoperative pathways, including Enhanced Recovery After Surgery (ERAS) recommendations for esophagectomy, are explicitly designed to reduce morbidity and improve recovery quality, and they provide an evidence-informed framework consistent with the complication reductions observed in our intervention group (28). Viewed through a health-system lens, complication reduction also functions as an efficiency lever because fewer adverse events translate directly into lower utilization of scarce inpatient and referral resources.

Beyond survival and complications, the model demonstrated clear patient-centered benefits. We used validated patient-reported outcome measures (PROMs), including the EORTC QLQ-C30 and the EC-specific QLQ-OES18, both of which have strong psychometric support for assessing quality of life and symptom burden in cancer populations, including patients with esophageal cancer (20, 22). The superior QoL trajectories and reduced EC-specific symptom burden observed in the intervention group suggest that the integrated model did not merely extend survival time but also improved survival quality. Notably, there is increasing high-level evidence that routine symptom monitoring using PRO systems can improve clinical outcomes, including survival, in oncology settings—supporting the plausibility that embedding structured symptom surveillance and responsive supportive care into routine management could contribute to better outcomes in our context (30, 31).

The economic evaluation further supports scalability. Compared with routine management, the intervention reduced direct medical costs and indirect costs, increased QALYs, and lowered the cost per QALY. Although GDP-based thresholds (e.g., 1–3× GDP per capita) have historically been used as reference points in many cost-effectiveness discussions, they have also been criticized as overly blunt and potentially misleading for local decision-making; nevertheless, they remain widely cited and can be used cautiously as contextual benchmarks (32). From a methodological standpoint, when QALYs are derived from cancer QoL instruments, mapping approaches from EORTC QLQ-C30 to preference-based measures (e.g., EQ-5D utilities) have been developed and validated, providing a defensible pathway for economic evaluation when direct utility measurement is unavailable (33). Mechanistically, the cost savings in our model likely arose from three synergistic pathways: earlier diagnosis reducing expensive late-stage care, fewer complications lowering downstream inpatient costs, and reduced out-of-county care seeking lowering patient-borne indirect costs.

Our multivariable analysis also underscores the importance of comorbidity management. Prior literature indicates that comorbidity burden (e.g., Charlson Comorbidity Index–based measures) is associated with worse postoperative outcomes and survival in esophageal cancer cohorts (23, 24). In our study, a higher comorbidity burden emerged as an independent mortality risk factor, suggesting that future optimization of county-level integrated EC care should explicitly incorporate structured cardiometabolic/pulmonary optimization and multimorbidity pathways (prehabilitation, medication management, and tighter coordination with primary care) to further reduce complications and improve treatment tolerance.

While our findings are encouraging, several limitations should be acknowledged. First, this was a single-county study; generalizability to other settings requires multi-site validation. Second, the non-randomized design means residual confounding cannot be fully excluded despite adjustment. Third, follow-up was limited to three years; longer surveillance is needed. Fourth, potential reporting bias cannot be fully excluded given the non-randomized, single-center setting. Fifth, the cost-utility analysis would be strengthened by explicitly reporting uncertainty (e.g., bootstrap confidence intervals) and clearly specifying the utility mapping approach (33).

Despite these limitations, our findings suggest that a county-adapted integrated full-cycle management model, anchored in a three-tier service network, MDT coordination, digital management tools, and standardized QoL monitoring, can substantially improve EC prevention and control performance in high-incidence county settings. With additional multi-site validation, strengthened implementation measurement, and further integration of comorbidity management, With multi-site validation, this approach may provide a scalable template for other resource-limited regions seeking to reduce the burden of EC and improve patient-centered outcomes. While resources vary across counties, the core components (three-tier network, digital platform, task-sharing) are adaptable to local conditions.

## 5 Conclusions

We developed a county-adapted integrated ‘prevention–screening–treatment’ model with full-cycle closed-loop management for EC. In a controlled evaluation, the model was associated with improved care processes, clinical outcomes, quality of life, and cost-utility compared with routine county management. With further multi-site validation and continued digital optimization, this model has potential to inform standardized county-level strategies for EC prevention and control.

## Data Availability

The original contributions presented in the study are included in the article/supplementary material. Further inquiries can be directed to the corresponding author.
